# Temperature dependency of excitonic effective mass and charge carrier conduction mechanism in CH_3_NH_3_PbI_3−x_Cl_x_ thin films

**DOI:** 10.1038/s41598-021-90247-x

**Published:** 2021-05-24

**Authors:** A. M. M. Tanveer Karim, M. K. R. Khan, M. S. Hossain

**Affiliations:** 1 Department of Physics, Rajshahi University of Engineering & Technology, Rajshahi, 6204 Bangladesh; 2 Department of Physics, University of Rajshahi, Rajshahi, 6205 Bangladesh

**Keywords:** Physics, Condensed-matter physics

## Abstract

In this paper we explain the temperature dependence of excitonic effective mass and charge carrier conduction mechanism occurs in CH_3_NH_3_PbI_3−x_Cl_x_ thin films prepared by chemical dip coating (CDC), spray pyrolysis (Spray) and repeated dipping-withdrawing (Dipping). Hall Effect study confirmed that prepared CH_3_NH_3_PbI_3−x_Cl_x_ samples are p-type semiconductor having carrier concentration of the order of ~ 10^16^ cm^−3^. The charge carrier mobility, mean free path and mean free life time were found to decrease with increasing temperature due to polaronic effect. The excitonic effective mass is estimated to (0.090–0.196)m_e_ and excitonic binding energy (15–33) meV, well consistent with Wannier-Mott hydrogenic model and the nature of exciton is likely to be Mott-Wannier type. From electrical measurement, it was observed that charge carrier conduction in CH_3_NH_3_PbI_3−x_Cl_x_ is governed by migration of ${\mathrm{I}}^{-}$ and CH_3_N ${\mathrm{H}}_{3}^{+}$ vacancies and vacancy-assisted diffusion processes depending on temperature.

## Introduction

Now-a-days, methylammonium lead halide perovskites (MLHP) are regarded worldwide as an emerging material class in the field of photovoltaic and optoelectronics. The typically chemical formula of MLHP is AMX_3_, where A is the organic/inorganic cation (CH_3_NH_3_), M is a heavy metal cation (Pb) and X is halide anions (I). When X site is replaced by another halogen element such as chlorine (Cl) into AMX_3_, it converts to mixed halide perovskites CH_3_NH_3_PbI_3−x_Cl_x_. This mixed halide perovskites exhibit semiconducting nature with some exciting properties for photovoltaic and optoelectronic device applications. The single-junction solar cell architecture, based on CH_3_NH_3_PbI_3−x_Cl_x_ perovskite, shows power conversion efficiency of 25.5%^1^. Besides, CH_3_NH_3_PbI_3−x_Cl_x_ thin film shows multi-colored emission^2–5^, second harmonic generation or frequency doubling^5^, bimolecular recombination^6^, trap-assist recombination^7^, strong tunable interband transitions^8^, and ambipolar charge transport^9^. However, mixed halide perovskite suffers a compositional instability due to halide (I, Br) segregation^10^. But a very small addition of Cl into (I/Br) lattice restrains the halide phase segregation, improves the charge carrier mobility, carrier lifetime and stability of solar cells without changing the crystallographic domain size^11^. These properties are directly correlated with the temperature dependent charge transport behavior in CH_3_NH_3_PbI_3−x_Cl_x_ and expected to improve device configuration compared to (I/Br) composition.

Up to now, multiple approaches such as transient terahertz (THz) spectroscopy^6,12^, combination of time-resolved terahertz with optical transient reflection spectroscopy (TRTS-TR)^13^, photoluminescence quenching (PLQ)^14^, time resolved electro-absorption spectroscopy^15^, time-of-flight (TOF) photoconductivity^16^ and Hall effect measurement^17^ have been carried out to explore the transport properties of lead halide perovskites. These pioneer studies reveal the presence of non-Langevin charge carriers^6^, strong back-scattering on free carrier dynamics^12^, existence of higher hole mobility compared to electron mobility^13^, large electron–hole diffusion lengths (˃1 μm)^14^, electric field assisted charge carrier seperation^15^, grain size dependent mobility^16^, and influence of self-doping in carrier concentration^17^. Despite the advancement in deep-seated perceptive in transport behavior, a lot of issues such as charge-carrier type, the mechanisms for restraining carrier mobility, origin of electron–hole recombination rates, donor or acceptor ionization energy, position of Fermi level, effective mass of charge-carriers, grain boundary barrier height, carrier mean free path-life time in CH_3_NH_3_PbI_3−x_Cl_x_ still perplexes the researchers. It is well known that the mixed halide perovskite undergoes a phase transition from orthorhombic-tetragonal-cubic at 160 K and in between (315–330) K, respectively^18,19^. However, the study of charge carrier dynamics is limited to tetragonal phase while it is also important to explore such properties for cubic phase above room temperature (RT).

Recently, it is reported that charge carries generate self-induced rotation of the organic part (CH_3_N ${\mathrm{H}}_{3}^{+}$), which polarizes lead halide perovskite lattice to form quasi-particle polarons^20–25^. The conception of polaron is used to explain intrinsic electrical properties because it describes the interaction between charge carriers and phonons. The interaction of charge carriers with the organic part has significant influence on the effective mass, temperature and magnetic field dependent carrier transport and electrical conductivity of the mixed halide perovskite material.

In our previous work we report the characterization of nano-crystalline CH_3_NH_3_PbI_3−x_Cl_x_ thin films prepared on glass substrate by chemical dipping-withdrawing (CDC)^2^, spray pyrolysis (Spray)^5^ and repeated dipping-withdrawing (Dipping)^3^ techniques in an ambient atmosphere. Our study confirmed the presence of tetragonal and mixed cubic-tetragonal phase in CH_3_NH_3_PbI_3−x_Cl_x_ thin film. The film morphology was composed with spherical, rod, cuboid and polyhedral like crystal grains of sizes 100 nm to 2 μm^2,3,5^. However, this article concentrates on the charge carrier transport of CH_3_NH_3_PbI_3−x_Cl_x_ thin films using Hall Effect and dc-electrical conductivity measurement as a function of temperature, ranging from RT to 378 K. The carrier concentration, mobility, excitonic effective mass, excitonic binding energy, Fermi level, grain boundary barrier height and mean free life time of charge carriers are calculated from Hall Effect study. Furthermore, activation energies in CH_3_NH_3_PbI_3−x_Cl_x_ thin films are exposed from electrical study. The effect of polarons on charge carrier dynamics and possible carrier conduction mechanism in CH_3_NH_3_PbI_3−x_Cl_x_ thin films has been discussed. Therefore, in-depth study of charge carrier transport and conduction mechanism above RT for CH_3_NH_3_PbI_3−x_Cl_x_ may provide a broader impact in the arena of perovskite research.

## Results

### Temperature dependent Hall effect study

**Figure 1:**
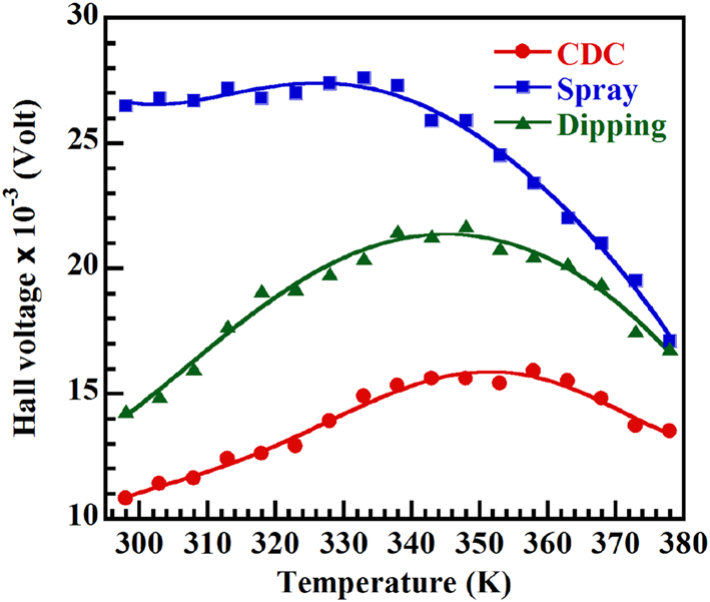
Hall voltage of CH_3_NH_3_PbI_3−x_Cl_x_. Variation of Hall voltage at constant magnetic field of 9.815 KG with temperature for CH_3_NH_3_PbI_3−x_Cl_x_ thin films using Hall measurement setup.

Temperature dependent Hall voltage measurement is done at a constant magnetic field of 9.815 KG and the variation of Hall voltage is shown in Fig. 1. From the Hall Effect study Hall mobility (µ_H_) and carrier concentration (n_c_) have also been calculated.

From Fig. 1 it is seen that the Hall voltage is positive and increased up to a certain temperature after which it decreases all through the measured temperature range for all samples. The positive sign of Hall voltage indicates that CH_3_NH_3_PbI_3−x_Cl_x_ is a p-type material. It is reported that a lot of Pb, CH_3_NH_3_, I and Cl vacancies present in the films^17,26,27^ as because precursor solution was prepared by dissolving CH_3_NH_3_I and PbCl_2_ at a molar ratio 3:1. However, Pb and CH_3_NH_3_ vacancies play the vital role for p-type conductivity of prepared CH_3_NH_3_PbI_3−x_Cl_x_ thin films because of the lower formation energy of Pb and CH_3_NH_3_ compared to halogen vacancies.

**Figure 2:**
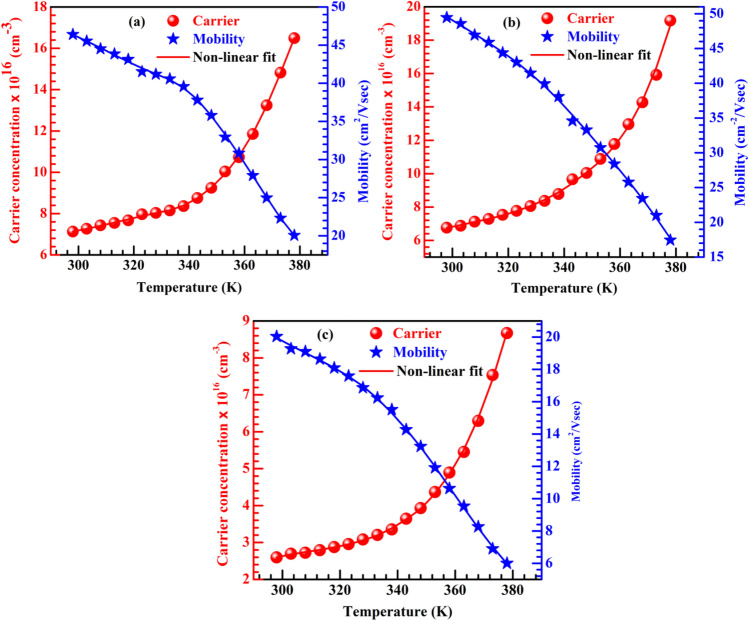
Charge carrier transport of CH_3_NH_3_PbI_3−x_Cl_x_. Variation of carrier concentration (left scale) and mobility (right scale) with temperature at a constant magnetic field of 9.815 KG for (**a**) CDC, (**b**) spray and (**c**) dipping deposited CH_3_NH_3_PbI_3−x_Cl_x_ thin films.

The temperature dependent carrier concentration (left scale) and mobility (right scale) for (a) CDC, (b) spray and (c) dipping deposite CH_3_NH_3_PbI_3−x_Cl_x_ thin films are shown in Fig. 2. The carrier concentration and mobility of dipping deposited sample is found almost half of the CDC and spray deposited samples. However, RT mobilities are found higher for all our samples than previously reported works^6,14,16,19,28–32^. Table 1 shows a comparison of RT mobilities for perovskite CH_3_NH_3_PbI_3−x_Cl_x_ obtained in different studies. It is reported that the breaking of inversion symmetry generates Rashba effect^33^ which enhance longitudinal optical phonon scattering by displacing the organic part or Pb-halogen bending or stretching and limits the increase of charge-carrier mobility at room temperature.

**Table 1:** The RT Hall carrier concentration (n_c_) and mobility (μ_H_) of CDC, spray and dipping deposited CH_3_NH_3_PbI_3−x_Cl_x_ thin films.

Perovskites	Measurement	n_c_ (cm^−3^)	μ_H_ (cm^2^/V-s)	Ref.
CH_3_NH_3_PbI_3−x_Cl_x_ thin films	Hall	7.1 × 10^16^	46	CDC This work
	Hall	6.8 × 10^16^	49	Spray This work
	Hall	2.6 × 10^16^	20	Dipping This work
	PLQ	–	2.1	^14^
	THz	–	11.6	^6^
	THz	1.0 × 10^15^	33	^28^
	THz	–	27	^29^
	Microwave		27	^30^
CH_3_NH_3_PbI_3_ single crystals	Hall	(9 ± 2) × 10^9^	105	^34^
	Hall	(1.0–1.2) × 10^11^	80–95	^25^
CH_3_NH_3_PbI_3_ thin films	THz	–	8	^6^
	THz		35	^19^
	THz	–	20	^31^
	TOF	–	0.25	^16^
	Microwave	–	30	^30^
	Microwave	–	29	^32^

The carrier concentration and mobility can be affected by both magnetic field and temperature but the effect of temperature is dominant. In this study, the carrier concentration of all CH_3_NH_3_PbI_3−x_Cl_x_ samples is found to increase exponentially (Fig. 2) with increasing temperature but mobility shows completely an opposite trend. Such behavior can be explained as: the presence of Pb, CH_3_NH_3_ and halogen vacancies in mixed halide perovskite creates free dipolar polarons by inducing a rotational re-organization of organic CH_3_NH_3_ dipoles^20^; such polarons can migrate between the inorganic site of perovskite structure and lower the electron–hole recombination. Furthermore, sub-lattice thermal vibrations of inorganic octahedral have also an effect on carrier concentration and mobility in perovskite samples. Both the reorientation of organic site and sub-lattice thermal vibration enhance the localized charge carriers which introduce a dynamic force for the movement of charges with increasing temperature. Therefore, a polarization potential of polaron originated by enhanced localization suppresses carrier velocity and reduce the mobility of CH_3_NH_3_PbI_3−x_Cl_x_ samples. Such a decrease of mobility with increasing temperature is reported^19^ measured by ultrafast spectroscopic method at temperature range -200 °C to 100 °C.

### Effective mass, excitonic binding energy and Fermi level calculation

**Figure 3:**
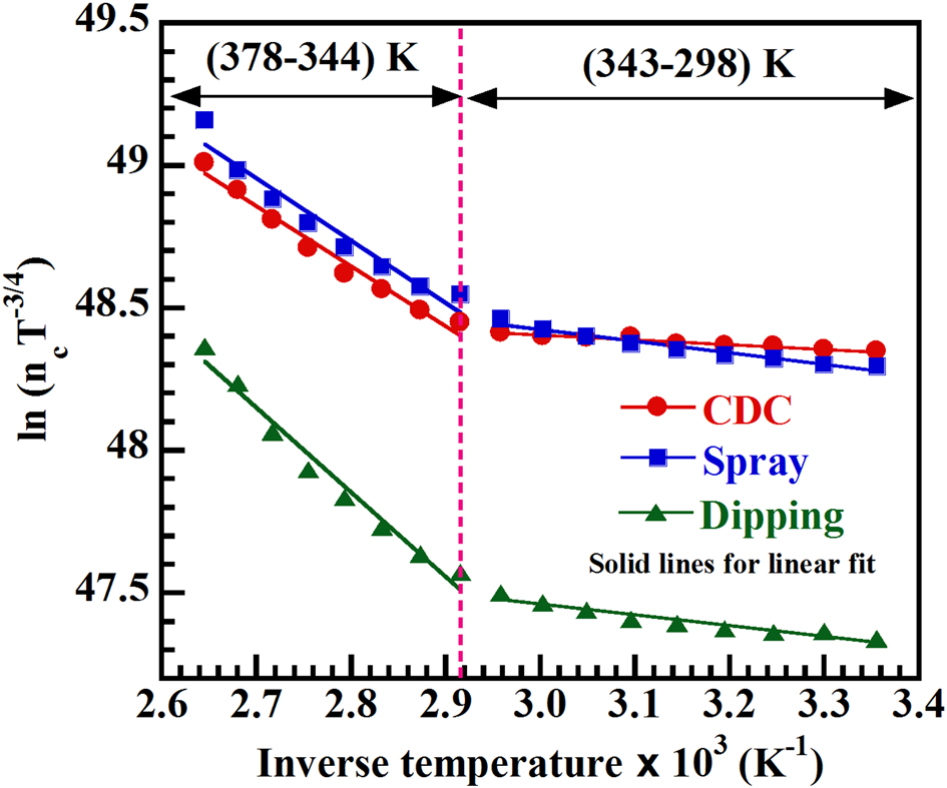
Diagram for calculating acceptor ionization energy. ln(n_c_T^−3/4^) vs inverse temperature graph for CDC, spray and dipping deposited CH_3_NH_3_PbI_3−x_Cl_x_ thin films.

The acceptor ionization energy, Fermi level and effective mass of charge carriers have been calculated from the temperature dependent Hall voltage measurement. The temperature dependent carrier concentration in the presence of a constant magnetic field can be represented by following equations,

1 $$ {\text{n}}_{{\text{c}}} = \left( {{\text{p}}_{0} {\text{N}}_{{\text{a}}} } \right)^{\frac{1}{2}} \exp \left( { - \frac{{{\text{E}}_{{\text{a}}} }}{{2{\text{K}}_{{\text{B}}} {\text{T}}}}} \right) $$

and

$$ {\text{p}}_{0} = \, 2\left( {\frac{{2\pi {\text{m}}_{{\text{h}}}^{*} {\text{K}}_{{\text{B}}} {\text{T}}}}{{\hbar^{2} }}} \right)^{\frac{3}{2}} $$

where E_a_ is the acceptor ionization energy, N_a_ is the acceptor density, ${\mathrm{m}}_{\mathrm{h}}^{*}$ is the effective mass of hole, T is the absolute temperature, K_B_ is the Boltzmann constant and ħ is the reduced Planck constant. The value of E_a_ can be obtained from the slope of ln $\left({\mathrm{n}}_{\mathrm{c}}{\mathrm{T}}^{-\frac{3}{4}}\right)$ vs. 1/T graph (Fig. 3). From figure it is clear that the variation of carrier concentration with temperature can be defined by two discrete temperature regions (TR): TR-I (298–343) K and TR-II (344–378) K. In TR-II, the values of ln $\left({\mathrm{n}}_{\mathrm{c}}{\mathrm{T}}^{-\frac{3}{4}}\right)$ decrease linearly with inverse temperature, whereas, in TR-I, it decreases monotonically with lower slope compared to TR-II. The effective mass of hole ${\mathrm{m}}_{\mathrm{h}}^{*}$ can be found out using E_a_ as,

2 $$ {\text{E}}_{{\text{a}}} = \frac{{{\text{e}}^{4} {\text{m}}_{{\text{h}}}^{*} }}{{2\left( {4\pi \varepsilon \varepsilon_{0 } \hbar } \right)^{2} }} $$

where ε is the dielectric constant of the material and ε_0_ is the permittivity of free space. Several research groups used dielectric constant ε = 5.6 to 25.7 (for high to low frequency) and excitonic effective mass μ* = 0.1m_e_ for calculating excitonic binding energy of perovskite. However, in this study ε = 9 has been used for calculating ${\mathrm{m}}_{\mathrm{h}}^{*}$ according to hydrogenic model^35^. The values of ${\mathrm{m}}_{\mathrm{h}}^{*}$ are estimated to 0.16m_e_, 0.42m_e_, 0.38m_e_ (m_e_ is the rest mass of electron) in the TR-I and 2.16m_e_, 2.23m_e_, 3.04m_e_ in the TR-II for CDC, spray and dipping deposited samples, respectively. The estimated ${\mathrm{m}}_{\mathrm{h}}^{*}$ is found in good agreement with recently reported values^36,37^. Taking the effective mass of electron ${\mathrm{m}}_{\mathrm{e}}^{*}$ = 0.21m_e_ from theoretical calculations^38^, the excitonic effective mass μ* is estimated to (0.090–0.196)m_e_ using equation, 1/μ* = 1/${\mathrm{m}}_{\mathrm{e}}^{*}$ + 1/${\mathrm{m}}_{\mathrm{h}}^{*}$ and tabulated in Table 2. The estimated μ* in TR-I is close to ~ 0.104m_e_^35^, found for tetragonal phase (≥ 145 K). However, μ* in TR-II is found high because of non-parabolic nature of valance bands in CH_3_NH_3_PbI_3−x_Cl_x_. Theoretical study^39^ suggested that lead vacancies diminish anti-bonding atomic orbital overlap resulting in flatten of valance bands. Moreover, polaronic effect^40^ and strong hydrogen bonding due to van-der-Waals interactions^39^ may also increase μ^*^.

**Table 2:** The acceptor ionization energy (E_a_), acceptor density (N_a_), Fermi level (E_F_), excitonic reduced effective mass (μ*) and excitonic binding energy (E_b_) of charge carrier for CDC, spray and dipping deposited CH_3_NH_3_PbI_3−x_Cl_x_ thin films for TR-I (298–343) K and TR-II (344–378) K.

Samples	E_a_ (eV)		N_a_ × 10^16^ (cm^−3^)		E_F_ (eV)		μ*/m_e_		E_b_ (meV)	
	TR-I	TR-II	TR-I	TR-II	TR-I	TR-II	TR-I	TR-II	TR-I	TR-II
CDC	0.028	0.364	2.00	6.34	− 0.014	− 0.182	0.090	0.191	15	32
Spray	0.071	0.378	3.80	11.3	− 0.035	− 0.189	0.140	0.192	24	32
Dipping	0.065	0.512	0.19	96.7	− 0.032	− 0.256	0.135	0.196	23	33

The excitonic binding energy, E_b_ of all perovskite samples has been calculated using the equation,

3 $$ {\text{E}}_{{\text{b}}} = \frac{{{\text{m}}_{{\text{e}}} {\text{e}}^{4} }}{{2\left( {4\pi \epsilon _{0} \varepsilon \hbar } \right)^{2} }}\frac{{\mu ^{*} }}{{{\text{m}}_{{\text{e}}} }}\left( {{\text{eV}}} \right){\text{ }} \approx {\text{ }}13.56\frac{{\mu ^{*} }}{{{\text{m}}_{{\text{e}}} }}\frac{1}{{\varepsilon ^{2} }}\left( {{\text{eV}}} \right) $$

The excitonic binding energy is found (15–24) meV for TR-I and (32–33) meV for TR-II, which is well consistent according to Wannier–Mott hydrogenic model. This variation of E_b_ in TR-I and TR-II is due to temperature dependent polaronic effect of CH_3_NH_3_PbI_3−x_Cl_x_. The extending radius of the lowest bound state r^*^ is calculated to ascertain the nature of excitons in CH_3_NH_3_PbI_3−x_Cl_x_ using the equation, r^*^ = ε (m_e_/μ^*^) r_b_, where, r_b_ is the Bohr radius. The r^*^ is estimated to 5.29 nm, 3.40 nm, 3.52 nm in TR-I and 2.49 nm, 2.48 nm, 2.43 nm in TR-II for CDC, spray and dipping deposited samples. The value of r^*^ is larger than the lattice constants (either cubic or tetragonal) of CH_3_NH_3_PbI_3−x_Cl_x_ indicating that the exciton is weak and likely to be Mott–Wannier type.

The position of Fermi level E_F_ can be determined by knowing the value of E_a_, N_a_ and ${m}_{h}^{*}$ of the following equation

4 $$ {\text{E}}_{{\text{F}}} = - \frac{1}{2}{\text{E}}_{{\text{a}}} + { }\frac{1}{2}\left( {{\text{K}}_{{\text{B}}} {\text{T}}} \right){\text{ln}}\left[ {\frac{{{\text{N}}_{{\text{a}}} {\text{h}}^{3} }}{{2\left( {2{\pi m}_{{\text{h}}}^{*} {\text{K}}_{{\text{B}}} {\text{T}}} \right)^{\frac{3}{2}} }}} \right] $$

The values of E_a_, N_a_ and E_F_ for both higher and lower temperature regions are tabulated in Table 2. The parameters n_c_ and N_a_ are not equal but vary with temperature and film processing methods as well, indicates CH_3_NH_3_PbI_3−x_Cl_x_ is very sensitive to environment, chemical composition, growth parameters etc. The Fermi energy is found negative means E_F_ lies below the acceptor level.

### Grain boundary parameters and grain size calculations

**Figure 4:**
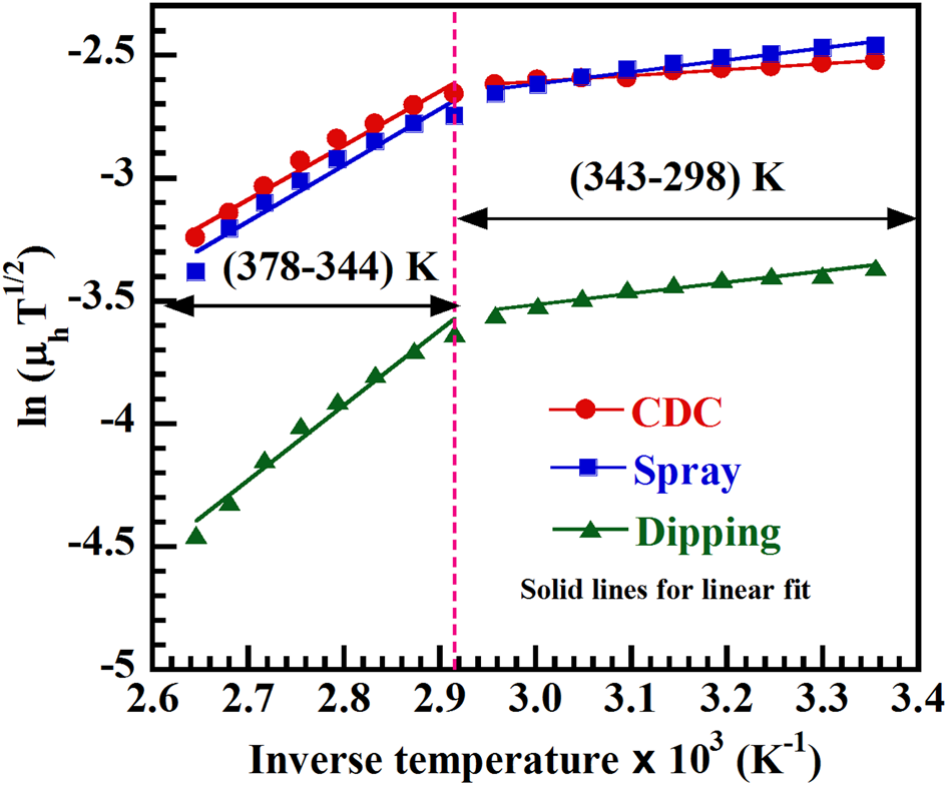
Figure for calculating grain boundary parameters. ln (μ_h_ T^1/2^) vs 1/T graph for CDC, spray and dipping deposited CH_3_NH_3_PbI_3−x_Cl_x_ thin films.

The transport properties of a polycrystalline semiconductor are generally influenced by grain boundary effect. According to grain boundary trapping model^41^, the trapping states create a depletion region in the grain and a potential barrier at the interface. In a semiconductor sample the relation between charge carrier mobility and grain boundary barrier height can be expressed as

5 $$ \mu_{{\text{h}}} = \, \mu_{0} {\text{T}}^{{ - \frac{1}{2}}} {\text{exp}}\left( {\frac{{ - \phi_{{\text{b}}} }}{{{\text{K}}_{{\text{B}}} {\text{T}}}}} \right){\text{ with}} $$

$$ \mu_{0} = \frac{{{{e\zeta }}}}{{\left( {8{\pi m}_{{\text{h}}}^{*} {\text{K}}_{{\text{B}}} } \right)^{\frac{1}{2}} }}, $$

where, ϕ_b_ is the grain boundary barrier height and $\upzeta$ is the grain size. The slop of ln(μ_h_T^1/2^) vs 1/T graph (Fig. 4) gives the barrier height ϕ_b_ and intercept will provide grain size $\upzeta .$

The barrier height and grain size of all samples have been estimated for two different temperature regions (TR-I, TR-II) and tabulated in Table 3. From table it is clearly seen that the formation of bigger grain size is favorable at low temperature (TR-I) for mixed halide perovskites.

**Table 3:** Grain boundary barrier height (ϕ_b_), grain size ($\zeta$), mean free path (L_m_), mean free life time (τ_m_) and activation energies (ΔE) for CDC, spray and dipping deposited CH_3_NH_3_PbI_3−x_Cl_x_ thin films.

Samples	ϕ_b_ (eV)		ζ (nm)		(L_m_)_avg_ × 10^–09^ (m)		(τ_m_)_avg_ × 10^–14^ (s)		ΔE (eV)		
	TR-I	TR-II	TR-I	TR-II	TR-I	TR-II	TR-I	TR-II	ΔE_I_	ΔE_II_	ΔE_III_
CDC	− 0.020	− 0.189	3.71	0.40	1.19	3.11	0.40	3.58	− 0.92	0.44	0.81
Spray	− 0.042	− 0.196	3.59	0.35	1.96	2.92	1.06	3.40	− 1.39	0.51	0.86
Dipping	− 0.039	− 0.264	1.98	0.10	0.76	1.28	0.39	1.74	− 0.29	0.47	0.57

### Mean free path and mean free time calculations

According to conventional Drude-Sommerfeld model, the carrier mean free path (L_m_) and mean free time (τ_m_) in CH_3_NH_3_PbI_3−x_Cl_x_ thin films can be calculated from the following equations

6 $$ {\text{L}}_{{\text{m}}} = {\upmu }_{{\text{H}}} \frac{{(3{\text{m}}_{{\text{h}}}^{*} {\text{K}}_{{\text{B}}} {\text{T}})^{\frac{1}{2}} }}{{\text{e}}} $$

7 $$ \tau_{{\text{m}}} = \frac{{{\upmu }_{{\text{H}}} {\text{m}}_{{\text{h}}}^{*} }}{{\text{e}}} $$

**Figure 5:**
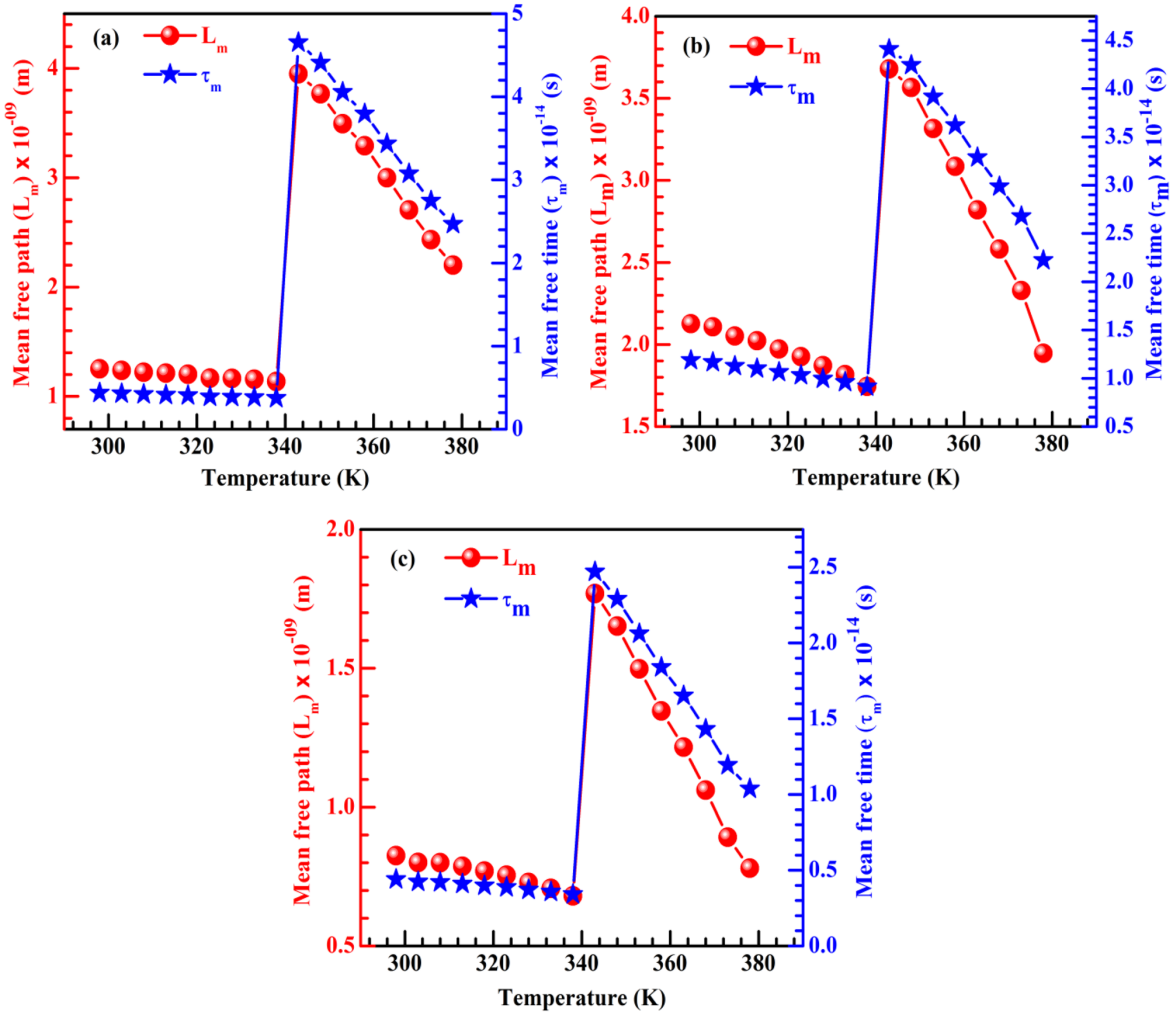
Mean free path and mean free time of CH_3_NH_3_PbI_3−x_Cl_x_. Mean free path and mean free time at different temperature region for (**a**) CDC, (**b**) spray and (**c**) dipping deposited CH_3_NH_3_PbI_3−x_Cl_x_ thin films.

The variation of L_m_ and τ_m_ with temperature for CDC, spray and dipping deposited CH_3_NH_3_PbI_3−x_Cl_x_ thin films are given in Fig. 5.

From figures it is seen that both L_m_ and τ_m_ are lower at TR-I compared to TR-II. The magnitudes of L_m_ and τ_m_ decrease with the increase of temperature except a sudden jump at around 340 K. The polaronic effect originated from rotational re-organization of CH_3_NH_3_ dipoles enhances trap assisted recombination with increasing temperature which reduces the carrier mean free path and mean free life time.

In the mixed halide perovskite, the transition from tetragonal to cubic phase occurs at temperature ~ 330 K^19,42^ due to the tilted inorganic PbI_6_ octahedral and disparity of organic CH_3_N ${\mathrm{H}}_{3}^{+}$ rotation. Such phase transition can modify the physical properties of mixed halide perovskites, though it does not cause remarkable change in optical and photovoltaic properties^43^. Recently, tetragonal to cubic phase transition is associated with the relative strength of the metal-halogen and hydrogen bonds as reported^44^. In our study, during the temperature dependent Hall measurement we have applied a magnetic field of ~ 1 T. This magnetic energy shifted the tetragonal to cubic phase transition at slightly higher temperature at (340–344) K.

### Conduction mechanisms and activation energies

**Figure 6:**
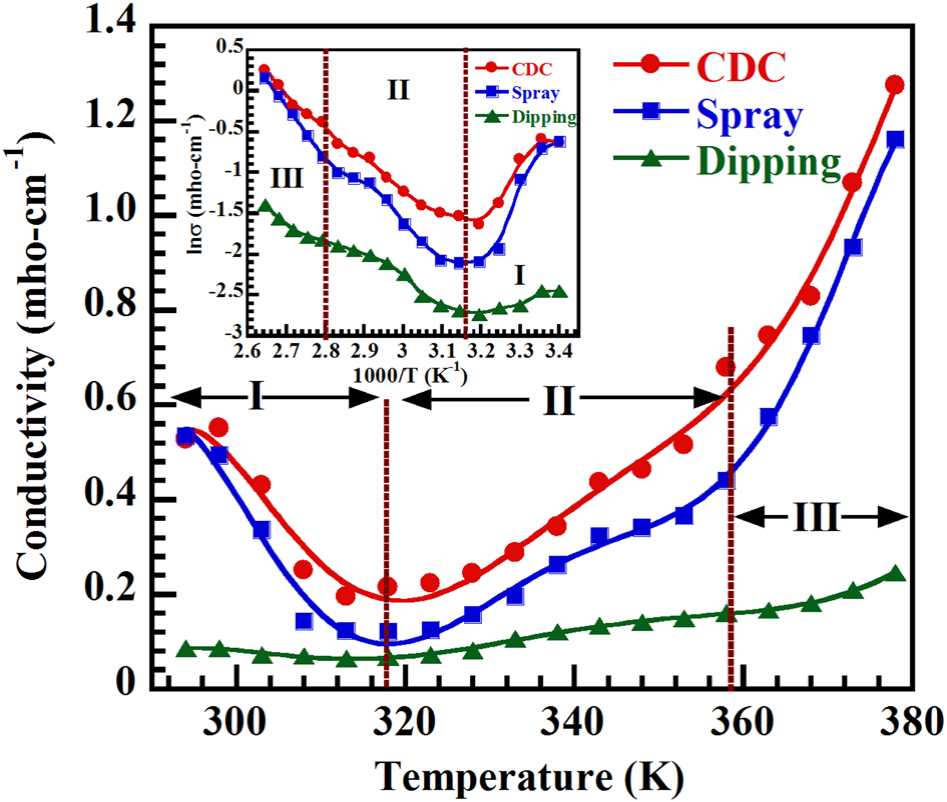
Electrical properties of CH_3_NH_3_PbI_3−x_Cl_x_. Temperature dependent dc-electrical conductivity of CH_3_NH_3_PbI_3−x_Cl_x_ thin films showing three distinct regions. Inset shows the variation of lnσ with 1000/T graph for calculating activation energy.

The electrical conduction process in CH_3_NH_3_PbI_3−x_Cl_x_ is rather complex. It is strongly dependent on temperature, vacancies or interstitial defects and activation energies of the materials. In general, CH_3_NH_3_PbI_3−x_Cl_x_ includes Pb, CH_3_NH_3_ and halogen vacancies as suggested from theoretical and experimental studies^45,46^. The low energy vacancies create a rotational re-organization of organic CH_3_NH_3_ dipoles resulting in free dipolar polarons in mixed halide perovskite^20^. Eames et al.^45^ proposed that these vacancies migrate from one site to the neighboring site due to low activation energy. They found activation energies involved 0.58 eV and 0.84 eV for migrating ${\mathrm{I}}^{-}$ and CH_3_N ${\mathrm{H}}_{3}^{+}$ vacancies, respectively. Futscher et al.^47^ also reported that the predicted activation energies for migration of ${\mathrm{I}}^{-}$ and CH_3_N ${\mathrm{H}}_{3}^{+}$ are (0.08–0.58) eV and (0.46–1.12) eV, respectively. Besides, grain boundary effect has an influence on carrier transport and the activation energies (0.18–0.27) eV were predicted for different grain size above 260 K^48^. In Fig. 6, it is clearly seen that there are three temperature regions where carriers are conducted through different conduction mechanisms. For further clarification, the activation energy was also calculated by Arrhenius equation which contains three separate activation energy terms,

8 $$ {\upsigma }_{{{\text{dc}}}} = {\upsigma }_{{\text{I}}} {\text{exp }}\left( {\frac{{ - \Delta {\text{E}}_{{\text{I}}} }}{{{\text{K}}_{{\text{B}}} {\text{T}}}}} \right) + {{ \upsigma }}_{{{\text{II}}}} {\text{exp }}\left( {\frac{{ - \Delta {\text{E}}_{{{\text{II}}}} }}{{{\text{K}}_{{\text{B}}} {\text{T}}}}} \right) + {\upsigma }_{{{\text{III}}}} {\text{exp }}\left( {\frac{{ - \Delta {\text{E}}_{{{\text{III}}}} }}{{{\text{K}}_{{\text{B}}} {\text{T}}}}} \right) $$

where, K_B_ is the Boltzmann constant, T is the absolute temperature, ${\upsigma }_{\mathrm{I}},$ ${\upsigma }_{\mathrm{II}}$ and ${\upsigma }_{\mathrm{III}}$ are pre-exponential factors and ΔE_I_, ΔE_II_ and ΔE_III_ are activation energies for region I, II and III, respectively. From the slope of lnσ vs 1000/T plot (inset of Fig. 6), the activation energies were calculated and tabulated in Table 3.

From Fig. 6, it is seen that dc-electrical conductivity (σ_dc_) decreases with increasing temperature in region I (294–317) K. The initial decrease is caused by the strong interactions in between organic–inorganic part and rotational disorder of methylammonium cation within perovskite structure. In addition, longitudinal optical phonon scattering may also decrease the conductivity with temperature in region I. The activation energy ΔE_I_ (Table 3) is found negative in region I, suggesting the Fermi level is pinned to the valance band of all samples. In region II (318–357) K, the dc-electrical conductivity increase slowly with increasing temperature and ΔE_II_ = 0.44, 0.51, 0.47 eV for CDC, spray and dipping deposited samples respectively, which indicates the migration of ${\mathrm{I}}^{-}$ vacancies to PbI_6_ octahedron edge^45–47^. In region III (358–378) K, σ_dc_ increases rapidly and ΔE_III_ ˃ ΔE_II_. The activation energies ΔE_III_ are found close to 0.84 eV for CDC and spray deposited and ΔE_III_ = 0.57 eV for dipping deposited samples. The rapid increase of σ_dc_ in region III occurs because of diffusion or migration of CH_3_N ${\mathrm{H}}_{3}^{+}$ in addition to ${\mathrm{I}}^{-}$ vacancies to PbI_6_ octahedra or neighbor central vacant site^45–47^. Therefore, the conduction processes are attributed to the migration of ${\mathrm{I}}^{-}$ and CH_3_N ${\mathrm{H}}_{3}^{+}$ suggesting the existence of ionic transport in CH_3_NH_3_PbI_3−x_Cl_x_. Moreover, the concentrations of charge carriers (majority holes) which are activated with acceptor ionization energy (E_a_) favorable for the transport across grain boundaries of CH_3_NH_3_PbI_3−x_Cl_x_ samples.

## Discussion

In summary, the charge carrier conduction mechanism of CH_3_NH_3_PbI_3−x_Cl_x_ thin films have been evaluated as a function of temperature ranging from room temperature to 378 K. Hall measurement study confirms that the prepared CH_3_NH_3_PbI_3−x_Cl_x_ films are p-type semiconductor with carrier concentration of the order of ~ 10^16^ cm^−3^. The carrier mobility found to decrease with increasing temperature due to polaronic effect. At RT, the mobilities for CDC, spray and dipping deposited samples are 46, 49 and 20 cm^2^V^−1^ s^−1^, respectively. The excitonic effective mass (μ^*^) is estimated to be (0.090–0.196)m_e_ and excitonic binding energy (E_b_) varies from (15–33) meV, suggesting Mott-Wannier type exciton. Additionally, the grain boundary barrier height, mean free path and mean free life time of charge carriers have also been evaluated. Electrical study reveals that multi-conduction processes are involved in CH_3_NH_3_PbI_3−x_Cl_x,_ which is governed by the migration of vacancies, vacancy-assisted diffusion of mobile ions and accumulation of holes across grain boundaries.

## Methods

### Synthesis

Lead halide perovskite solution for fabricating CH_3_NH_3_PbI_3−x_Cl_x_ thin films was prepared using the following procedure. Methylamine (CH_3_NH_2_) solution was mixed with hydroiodic acid and ethanol at room temperature. The solution was perturbed continuously by stirring with a magnetic stirrer at a constant speed in an ice bath for 2 h. The color of CH_3_NH_3_I solution transforms from red to transparent and heated at 100 °C for 4 h in a furnace to achieve white-colored CH_3_NH_3_I powder. Finally, CH_3_NH_3_I and lead(II) chloride powders were dissolved in anhydrous N,Ndimethylformamide at a molar ratio 3:1 to achieve a mixed halide perovskite precursor solution.

CH_3_NH_3_PbI_3−x_Cl_x_ thin films have been fabricated at ambient atmosphere by three different methods. These methods are (a) Chemical dip-coating (CDC) (b) Spray pyrolysis (spray) and (c) Repeated dipping-withdrawing (dipping). The details of preparing CH_3_NH_3_PbI_3−x_Cl_x_ solution and different steps which have been made in these methods to deposit CH_3_NH_3_PbI_3−x_Cl_x_ thin films have been reported elsewhere^2,3,5^.

### Characterization

Temperature dependent Hall voltage was measured at constant magnetic field of 9.815 KG using the conventional van-der-Pauw method. The magnetic field used for the study of Hall Effect was provided by electromagnets designed and produced by Newport instruments Ltd. England. The circuit arrangement for temperature dependent Hall Effect measurement is shown in Fig. 1S. A dc voltage from the power supply unit was applied in order to flow the sample current through the specimen. Current and voltages were measured by a digital electrometer and a digital multi-meter. A voltage stabilizer was used so that a steady supply of current without fluctuation was maintained through the magnet. A special designed heater was used to vary temperature. The variation of temperature is controlled by a variac and measured by a chromel–alumel thermo-couple connected with a digital multi-meter.

The resistivity of all samples was measured by van-der-Pauw method. Experimental setup of the van-dar-Pauw’s specimen to measure the resistivity with varying temperature is shown in Fig. 2S. The voltage and current of all samples were measured for different temperatures. The sample is fixed to a sample holder which is placed on a specially designed heater to vary temperature. The conductivity and activation energies of prepared samples have been calculated using resistivity data. The experimental details of temperature dependent Hall Effect measurement and resistivity measurement are shown in supplementary section.

## Supplementary Information

Supplementary Information.
