# Supplementary material for: Temperature dependency of excitonic effective mass and charge carrier conduction mechanism in CH3NH3PbI3−xClx thin films
Source: Sci Rep. 2021 May 24;11:10772. doi: 10.1038/s41598-021-90247-x (PMC8144584; doi:10.1038/s41598-021-90247-x)
Supplement: Supplementary file 1 — Supplementary Information. [file 41598_2021_90247_MOESM1_ESM.docx]

**Temperature dependency of excitonic effective mass and charge carrier conduction mechanism in CH_3_NH_3_PbI_3-x_Cl_x_ thin films**

A. M. M. Tanveer Karim^1*^, M. K. R. Khan^2*^, M. S. Hossain^1^

^1^Department of Physics, Rajshahi University of Engineering & Technology, Rajshahi-6204, Bangladesh

^2^Department of Physics, University of Rajshahi, Rajshahi-6205, Bangladesh

*Corresponding authors

E-mail: [tanveer@phy.ruet.ac.bd](mailto:tanveer@phy.ruet.ac.bd), [tanveerruphy@gmail.com](mailto:tanveerruphy@gmail.com,), [mfkrkhan@yahoo.com](mailto:mfkrkhan@yahoo.com)

**Temperature dependent Hall measurement**

Temperature dependent Hall Effect was measured using the conventional van-der-Pauw method. The circuit arrangement for temperature dependent Hall Effect measurement is shown in Fig. 1S.


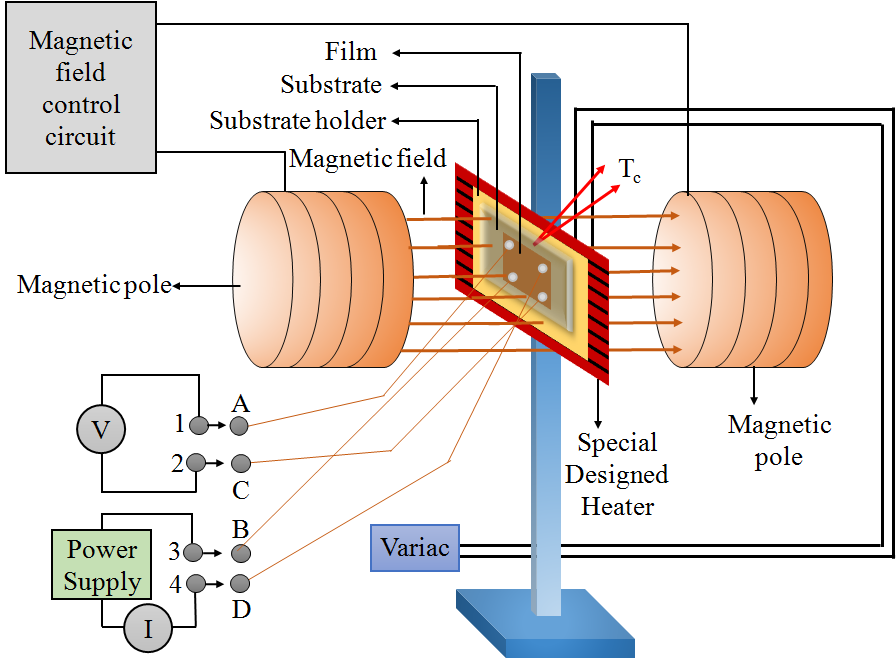


**Fig. 1S Hall Effect measurement.** Experimental setup for temperature dependent Hall Effect measurement using the conventional van-der-Pauw method. 1, 2, 3 and 4 are meter terminals and A, B, C and D are the film terminals. A dc voltage from the power supply unit was applied between the contacts A and C in order to flow the sample current through the specimen. The Hall voltage was obtained between the contacts B and D by applying constant magnetic field (of the order of 9.815 KG) normal to the film surface. V and I represent the voltmeter and ammeter, respectively. T_c_ is the digital multi-meter acting as thermocouple.

In the present work, the voltage V_1_ and V_2_ were measured where V_1_ is the potential across voltage probes B and D when the magnetic field B is zero and V_2_ is the potential when a steady magnetic field is applied. The change in resistance ΔR_AB,CD_ was calculated by using the relation:

ΔR_AB,CD_ = $\frac{V_{2}-V_{1}}{I_{\mathrm{AC}}}$ (1)

where I­_AC_ is the current flowing through the specimen between the contacts A and C. The carrier concentration and Hall co-efficient were calculated by using the relations

n= $\frac{1}{eR_{H}}$ (2)

R_H_ = $\frac{{\Delta R}_{AB.CD}\times t}{B}$ $\times{10}^{8}$ cm^3^/coul (3)

where the magnetic field B is in Gauss and the film thickness t is in cm.

**Measurement of resistivity and activation energy**

Van-der-Pauw method is one of the standard and widely used techniques for the measurements of resistivity of thin films. Fig. 2S shows the experimental setup of the Van-dar-Pauw’s specimen to measure the resistivity with varying temperature. Four small contacts A, B, C, D and the points 1, 2, 3 and 4 indicate the terminals of the electrometer for the measurement of current and voltage, respectively. The voltage and current of the sample were measured for different temperatures. The sample is fixed to a sample holder which is placed on a specially designed heater to vary temperature.

The resistivity ρ and hence, the conductivity σ was calculated using the equation

ρ = 2.265 t (R_AC.BD_+R_AD.BC_) Ohm-cm (4)

Where, t is thickness of the film, R_AC.BD_ = V_BD_/I_AC ,_ R_AD.BC_ = V_BC_/I_AD._

The activation energy ΔE of samples was calculated from the slope of lnσ versus 1/T plot based on the equation

ΔE = -lnσ/(1/T)×2K_B_ (5)


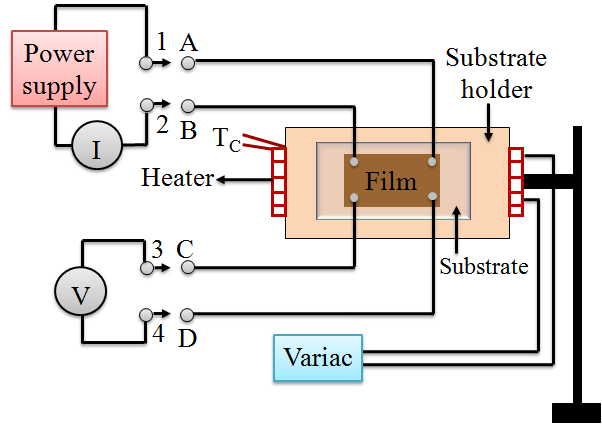


**Fig. 2S Resistivity measurement setup.** Schematic diagram for the measurement of temperature dependent resistivity of CH_3_NH_3_PbI_3-x_Cl_x_ thin films. 1, 2, 3 and 4 are meter terminals and A, B, C and D are the film terminals. Arrows indicate commutating switches. V and I represent the voltmeter and ammeter, respectively. T_c_ is the digital multi-meter acting as thermocouple.
